# Motivators of couple HIV counseling and testing (CHCT) uptake in a rural setting in Uganda

**DOI:** 10.1186/s12889-017-4043-z

**Published:** 2017-01-23

**Authors:** Victoria Nannozi, Eric Wobudeya, Nicholas Matsiko, Jacqueline Gahagan

**Affiliations:** 1 Makerere University Joint AIDS program, P. O. Box 7071, Kampala, Uganda; 2 Mulago National Referral Hospital, P. O. Box 7051, Kampala, Uganda; 3 Gender & Health Promotion studies unit (GAHPS unit), Dalhousie University School of Health & Human Performance, 6230 South Street, Halifax, NS B3H 3J5 Canada; 4 Joint Clinical Research Center, P. O. Box 10005, Kampala, Uganda

**Keywords:** Couple HIV counseling and testing, Motivate, Uptake

## Abstract

**Background:**

Couple HIV Counseling and Testing (CHCT) is one of the key preventive strategies used to reduce the spread of HIV. In Uganda, HIV prevalence among married/living together is 7.2% among women and 7.6% among men. CHCT can help ease disclosure of HIV-positive status, which in turn may help increase opportunities to get social support and reduce new infections. The uptake of CHCT among attendees of health facilities in rural Uganda is as high as 34%. The purpose of this study was to explore the motivators of CHCT uptake in Mukono district, a rural setting in Uganda.

**Methods:**

The study was conducted in two sub-counties in a rural district (Mukono district) about 28 km east of the capital Kampala, using a descriptive and explorative qualitative research design. Specifically, we conducted focus group discussions and key informant interviews with HIV focal persons, village health team (VHT) members, religious leaders and political leaders. We also interviewed persons in couple relationships. Data was analysed using NVivo 8 software. Ethical clearance was received from the Mengo Hospital Research Review Board and from the Uganda National Council of Science and Technology.

**Results:**

The study was conducted from June 2013 to July 2013 We conducted 4 focus group discussions, 10 key informant interviews and interviewed 53 persons in couple relationships. None of the participants were a couple. The women were 68% (36/53) and 49% (26/53) of them were above 29 years old. The motivators of CHCT uptake were; perceived benefit of HIV testing, sickness of a partner or child in the family and suspicion of infidelity. Other important motivators were men involvement in antenatal care (ANC) attendance and preparation for marriage.

**Conclusion:**

The motivators for CHCT uptake included the perceived benefit of HIV testing, sickness of a partner or child, preparation for marriage, lack of trust among couples and men involvement in antenatal care. Greater attention to enhancers of CHCT programming is needed in trying to strengthen its uptake.

**Electronic supplementary material:**

The online version of this article (doi:10.1186/s12889-017-4043-z) contains supplementary material, which is available to authorized users.

## Background

In 2015 there were about 2 million new HIV infections globally, with Southern and Eastern Africa accounting for about 1 million (50%) of them [1]. In Uganda, the prevalence of HIV among persons aged 15 to 49 years increased from 6.4% in 2005/06 to 7.3% in 2011 [2] while HIV incidence among adults increased from 134,634 in 2011 to 139,178 in 2012 [3]. Non-disclosure of HIV status among couples [4] in addition to unknown partner’s HIV status, illiteracy, polygamy, resistance to sexual behavior change are some key socio-cultural factors contributing to most of new infections [5]. A prospective cohort study in Zambia showed an HIV seroconversion rate of 8.5 per 100 person years among HIV discordant couples [6]. Another study in Southern and Eastern Africa showed that 49% of the HIV affected couples are discordant [7]. In Uganda, about 3% of couples are discordant and it is more common where the man is older by at least 10 years [2]. The HIV prevalence among women married/living together persons is 7.2% while that in men married/living together is 7.6% [2]. Previous research has showed that male partners are more likely to bring HIV infection into a couple relationship, while females become infected at a higher rate [8].

Based on the observed high proportion of discordant couples with potential for new HIV infections among them, the World Health Organization issued guidelines for Couple HIV Counseling and Testing (CHCT) in 2012 aimed at people who are in a sexual relationship and wish to test together and mutually disclose their results [9]. Couple based intervention and prevention studies reduce HIV incidence among HIV-negative sex partners and viral load among HIV-positive partners [10]. However, several barriers to CHCT have been reported including fear of a positive test [11], mistrust among couples [12] and limited men involvement [12–14]. In 2009 the government of Uganda released the National CHCT and communication strategy with the purpose to increase CHCT uptake and reduce new HIV infections [15].

The uptake of CHCT among attendees of health facilities in rural Uganda is up to 34% [16, 17]. A figure that has remained low but what motivates these few couples to test? The literature on motivators for CHCT uptake is limited. Some reported motivators of CHCT uptake include prior couples’ HIV counseling and Testing (HCT) and prior discussion of HIV testing with a partner [13]. A study in Uganda showed that prior individual knowledge of HIV status, exposure to promotional messages and prior discussion with a partner [18] were associated with CHCT uptake. These studies were carried out in various regions with different HIV sero-prevalence and background socio-economic factors using quantitative methods. Our study was conducted in a rural setting under routine programmatic health care service delivery using qualitative methods. We aimed at exploring the motivators of CHCT uptake in Mukono district in Uganda.

## Methods

### Study design

This study employed a descriptive, exploratory qualitative research design in keeping with Maxwell JA [19]. This qualitative approach allowed an in-depth understanding of the perceived benefits and motivators to CHCT uptake in a rural setting.

### Study setting

The study was conducted in Mukono district, a rural district in Uganda from June 2013 to July 2013. Mukono district is in Central Uganda, 28 km east of the capital Kampala. According to the population projection from the 2002 National census, Mukono district had a population of 536,400 [20]. The HIV prevalence rate in this region is 12.5% among women and 8.4% among men aged 15 to 49, with about 11% of the couples being HIV sero-discordant [2]. The study was conducted in 2 sub-counties in Mukono; the Central division at Mukono Health Center IV, which has the highest CHCT uptake, and Kyampisi sub-county at Kyampisi Health Center III which has the lowest CHCT uptake. The CHCT approach at these facilities is through provider initiated counseling and testing.

### Study participants

we had participants at individual and focus group discussion levels. The participants were persons in couple relationships that had come to the health units for health care. The individual interviews provided individual level characteristics and responses while the FGD groups were used to get a collective view of group participants, while knowing that group dynamics can stimulate more ideas and facts. We included 53 participants aged 20 to 49 years that reported being in couple relationships, had tested for HIV and were residents of Mukono district. For this study, a couple relationship was defined as two adult persons in an ongoing sexual relationship who had lived together for at least 3 months [10]. We considered 3 months because it is the typical duration for HIV sero-conversion and has been used in previous studies [10]. The individuals were approached to join the study and those who met the eligibility criteria were consecutively enrolled. Enrollment continued until point of data saturation was reached where no new information was reported by the participants. Every individual interview took about 40 min. None of these individuals participated in the FGDs and key informant interviews.

We conducted 4 FGDs and each took about 60 min. Participants in the FGDs were all either male or female. Each FGD had 9 to10 participants. The local council chair moved door to door within the study area to invite participants; those who met the inclusion criteria and provided informed consent participated. FGDs occurred at times and in places where other meetings are usually held to convenience of the study participants.

Ten key informants were purposively selected by the Assistant District Health Officer. These included 2 religious leaders, 2 district local leaders, the district HIV focal person, and 5 village health team (VHT) leaders. The key informants were considered knowledgeable in the community dynamics. The VHTs are key resources in their villages for vital health information while the district HIV focal persons are the implementers of HIV Counselling and Testing policies in the district.

#### Data collection and Analysis

The purpose of the study was explained to all participants and they were invited to voluntarily participate in the study after providing informed consent. Data was collected using semi-structured interview guides for individual interviews (see Additional file 1) by the research assistant who ensured an open and collegial atmosphere for the interviews. Individual data collected included; personal characteristics, factors that influence CHCT, barriers, benefits and risks of not testing for HIV as a couple. For the FGDs we used an FGD guide (see additional file 2) while for key informants an interview guide (see Additional file 2) was used. The same guide themes were used for FGDs and key informant interviews. The themes were; benefits, risks, barriers, health worker influence and structures in place to motivate CHCT. Individual interviews data was presented as frequencies and proportions for individual HCT (Couples that tested as individuals) and couple HCT (individuals who tested as couples). All data collected in the local language Luganda was transcribed verbatim and translated into English. The Principal Investigator moderated the FGDs, while a research assistant wrote notes on the overall discussions. Audio recording of all the interviews was done with permission of participants. The interviews were conducted by two research assistants. All transcribed data was coded, analyzed and managed using NVivo 8 software. Data was triangulated across the three data sources: individual interviews, focus group discussions (FGDs), and key informant interviews.

## Results

None of the participants were a couple. The socio-demographic characteristics of the interviewed individuals are in Table 1.

**Table 1:** Characteristics of interviewed individuals in couple relationships in Mukono, 2013 (N = 53)

Characteristic		Individual HCT *n* (%)	Couple HCT *n* (%)
Gender	Female	23 (21)	13 (46)
	Male	6 (79)	11 (54)
Age group	18–29years	14 (48)	13 (54)
	≥30 years	15 (52)	11 (46)
Education^a^	Primary	14 (50)	10 (42)
	Post-primary	14 (50)	14 (58)
Occupation	Peasant	8 (32)	5 (18)
	Salaried	1 (4)	4 (14)
	Self employed	11 (44)	15 (54)
	Unemployed	5 (20)	4 (14)
Marriage type	Monogamous	19 (66)	23 (96)
	Polygamous	8 (28)	1 (4)
	Regular partner not co-habiting	2 (7)	0 (0)

^a^1 missing data

Among the 35 FGD participants; male 19/35 (54%), female 16/35 (46%). We found that of the 10 key informants interviewed, 3 (2 VHT members, and 1 District HCT focal person) had tested as couples though they were interviewed as key informants.

Five key themes emerged from the information got from study participants related to motivators of CHCT in a rural setting. These themes included the perceived benefits of support for HIV care, sickness of a partner, preparation for marriage, lack of trust among couples, male involvement in antenatal care (ANC) attendance.

### Perceived benefit of HIV testing

The respondents thought CHCT leads to early initiation into HIV care and treatment support and prevention for those who test negative.

*“…when a couple discovers that they are discordant at the point of testing they can be counseled on how they can live together without infecting the negative partner” Male FGD participant*

*“…know your HIV status and it gives you courage to take the drugs and others reduce the number of partners that they have” Male participant in FGD*

*“Such that in case we are HIV positive we can start on treatment” Female individual participant*

*“…sensitizations in the community by community counsellors.” Female individual participant*

### Sickness

Sickness in the family was an important contributor to CHCT uptake. Some reported that they were sickly and were encouraged to test for HIV with their partners.

*“I would fall sick frequently with fevers and diarrhea” Female individual participant*

In other incidences the partner or child was sick.

*“…my husband was sick” Female Individual participant*

*“*…*Thought we were HIV negative but when my child was sick we both tested and we were both HIV positive” Male individual participant*

### Preparation for marriage

Another theme that came out strongly as an enhancer to CHCT was the preparation for marriage.

*“we tested as couple when I wanted to marry my girlfriend” Male individual participant*

*“It’s only couples that intend to get married that test together…Male VHT member*

*“…what encourages CHCT is when they are getting married and the man wants to take a wife when they know their status” Female FGD participant*

### Mistrust among couples

Participants reported lack of trust for a partners HIV status enhances the likelihood of CHCT uptake.

*“me I disagree with the fact that couples test together because they trust one another, I think there is usually doubt whether the person is HIV positive or negative so test to rule out this” Male FGD participant*

*“Unfaithfulness in marriages brought about by rumours of infidelity” Local council Leader*

### Men involvement in ANC attendance

When couples present at the health unit, they are encouraged by the health care workers to test together. This mainly occurs in trying to reduce the likelihood of mother to child HIV transmission. The health care workers also encourage women who attend antenatal care to bring their husbands with them for CHCT.

*“…was pregnant and it was a requirement…” female individual participant*

*“……when they escort their wives during antenatal visits the wives are tested for HIV and when the men are asked is this your wife and you say yes you definitely have to be tested….” Male FGD participant*

## Discussion

This study set out to explore potential motivators of CHCT uptake in the rural district. Our findings indicate that the motivators for CHCT uptake included perceived benefit of HIV testing, persistent sickness in the family, and preparation for marriage, lack of trust among couples and men involvement in ANC attendance.

As indicated by our data, the perceived benefits of CHCT in case of having a positive HIV test was a key enhancer to CHCT uptake. Further, our participants thought it leads to early initiation into HIV care and treatment, risk reduction for the discordant couples and Prevention of Mother to child transmission (PMTCT). A study in Zambia exploring couple experiences revealed that some couples found it easier to access and remain in care owing to the support of their spouses [17]. In the same study the women found it easier to negotiate safe sex when discordant [17]. Another study, in Malawi, found that mutual encouragement was one of the motivators for CHCT among cohabiting couples [21].

Reduction in HIV incidence among HIV-negative sex partners and viral load among HIV-positive partners is a known benefits of CHCT interventions [10]. A study in Uganda found that uptake of voluntary counseling and testing (VCT) was directly related to assurance of linkage to ART care [22]. The perceived benefits of CHCT in our study were facilitated by the increased knowledge of CHCT. CHCT sensitization had occurred in the community by Local council leaders, civil society organizations and community counselors. A systematic review of strategies to increase HIV testing uptake found that health worker notifications increase HIV testing uptake in targeted communities. In a multi health disease campaign in rural Uganda, more individuals tested for HIV after 1 month of sensitization in the community [23]. Peer education is a well-known intervention that can increase knowledge and positive attitudes towards HIV/AIDS among adolescents [24].

Our study revealed that sickness in the family was a contributor toward CHCT uptake. Specifically, several participants reported that they repeatedly developed infections which prompted testing for HIV with their partners. In other incidences the partner or child was sick and this enhanced the need for testing. Despite this, there is limited published data on sickness as a determinant of couple HIV testing. Acceptance as high as 98% has been reported that among those offered routine HCT in two large hospitals in Uganda [25]. This also presents a key opportunity to receive CHCT.

Preparation for marriage was a strong enhancer of CHCT uptake. HIV testing is a prerequisite in many churches in Uganda before the wedding. This idea of HIV testing has extended to those who intend to commit to a serious relationship. A study carried out in Nigeria revealed that many youth have a positive attitude towards mandatory pre-marital CHCT [26].

We found that the lack of trust among couples enhanced CHCT uptake. Individuals in relationships where they are suspicions of an unfaithful partner are motivated to access CHCT. Testing as a couple is thought to build trust among couples as it helps rule out a lack of faithfulness. Previous reports suggest that mistrust is a greater motivator for CHCT where women suspect infidelity [21]. Another study found an opposing view in that men who perceived their marriages as unstable and distrustful, were not motivated to access couple testing because of the conflicts that could arise [12]. This findings suggests that mistrust may either be a barrier or enhancer of CHCT. Women will more probably insist on CHCT where they suspect infidelity and it may be the only subtle way to settle the fear of being HIV infected. On the other hand, most males will be unwilling to undergo CHCT most especially when they know about their marriages are unstable and they have extra-marital affairs.

Our findings show men involvement in ANC was a motivator for CHCT uptake. Similar findings have been reported by Okoli and others in Nigeria where 31% of respondents agreed that men involvement in ANC would promote CHCT uptake [27]. Despite this finding it has been reported that while interested in attending the ANC with their wives, still few men accept CHCT [28]. One of the barriers for testing as couples is fear of a positive HIV test [29] and have reported feeling as if ‘being taken to the police’ [18]. Fear of an HIV positive test is a documented barrier to CHCT uptake in our setting.

The qualitative exploratory nature of our study enabled us to gain an in-depth understanding of some of the underlying factors for CHCT uptake. However, some limitations to our study should be noted in moving CHCT research forward. Given that there were more women in the study population than men; our study population may not reflect a balanced gender community perspective. This is important given that we purposively recruited from individuals who accessed health care at the selected health units, which may suggest these individuals have high levels of health seeking behaviours.

## Conclusion

CHCT is an important public health intervention aimed at increasing awareness of testing and reducing the onward transmission of HIV. Our study conducted in a rural community in Uganda found that the motivators of CHCT uptake are; perceived benefit of CHCT, persistent sickness in the family, preparation for marriage, lack of trust among couples and men involvement in ANC attendance. Our findings indicate that there is a diversity of opinions and perspectives on motivators of CHCT uptake in this setting.

Notwithstanding our limitations, the findings of this study are important in informing community interventions to increase CHCT uptake. We think it is important to invest in interventions that the community already believes in and knows. In this study the identified motivators could be potential intervention targets for increased CHCT uptake. Consequently, the other benefits of CHCT such as disclosure of HIV status to marital partner, renewed commitment to marital relationship, uptake of and adherence to treatment and formation of new social networks [30] will also be realized. CHCT is also more likely to contribute to prevention of mother to child HIV transmission as mothers are more likely to receive early anti-retroviral medicines for themselves and their children [31]. Additional emphasis is needed on addressing the motivators for CHCT uptake in trying to improve access to and uptake of this important testing intervention.

## Additional files

Additional file 1: Interview guide for Individuals in couple relationships (DOC 43 kb)
Additional file 2: Key informant interview and FGD guide (DOC 25 kb)

## Abbreviations

ANC: Antenatal clinic
CHCT: Couple HIV Counselling and Testing
FGD: Focus group discussion
HCT: HIV Counselling and Testing
HIV: Human immune-deficiency virus
VHT: Village health team
