# Supplementary material for: Motivators of couple HIV counseling and testing (CHCT) uptake in a rural setting in Uganda
Source: BMC Public Health. 2017 Jan 23;17:104. doi: 10.1186/s12889-017-4043-z (PMC5259987; doi:10.1186/s12889-017-4043-z)
Supplement: Additional file 1: — Interview guide for Individuals in couple relationships (DOC 43 kb) [file 12889_2017_4043_MOESM1_ESM.doc]

**Interview guide for Individuals in couple relationships**

Dear Respondent,

We, a team from Mukono Local government, Health department are seeking to know the factors that influence the uptake of couple HCT in Mukono.

Your name has been randomly selected. If you consent to participate, you will be asked questions on economic, social and structural factors that influence the uptake of CHCT in Mukono.

Your responses will remain confidential and will only be known to the investigators. Blood specimens are not taken in this survey.

Participation in the study is entirely voluntary, you are free to refuse to participate or stop the interview at any point.

SECTION A:

**DEMOGRAPHICS**

Village______________________________Parish__________________________

Sub county___________________________ county __________________

**Please put a tick (√) inside the box that corresponds to the answer given**

1. Gender

|__|Male |__|Female

1. Age bracket

|__|15-24 years |__| 25-35 year |__|36-45years |__|Over 45 years

1. Highest level of education

|__|never went to school |__|A level |__|Primary Level |__|Certificate

|__|O level |__|Diploma |__| University

|__|Any other specify_______________________

1. Occupation

|__|Peasant |__|Housewife |__| Self employed |__|Salary employee

|__|farmer |__|Other specify______________________________

**SECTIONB: Social Factors and CHCT**

1. Do you have a partner at the moment?

|__|Yes |__|No

1. How would you describe your partnership?

|__| Married/Co-Habiting Monogamous |__| Married/Co-Habiting Polygamous

|__| Regular Sex Partner, Not Co-Habiting |__| Separated/Divorced

|__| Widowed |__| other specify_____________________

1. How long have you been in a relationship with this person?

|__|__|Years and |__|__|Months

1. Does your partner stay with you:

|__| The whole time |__| On weekends

|__| One/two weekend per month |__| Doesn’t stay with partner

|__| Other – specify___________________________________

1. Have you ever been tested for HIV?

|__| No |__| Yes

1. If yes were you tested with your partner as a couple

|__| No |__| Yes

1. If yes to question 6 when you were last tested as a couple?

|__|__|__| /|__|__|__|__| (MON/YYYY)

1. Where did you take your last test from?

|__| Public hospital |__| Public health centre |__| Private Hospital

|__| Private health centre |__| NGO community |__| Public health centre-community

|__| Blood service |__| Other specify_________________________________

**SECTION C: Social Factors and CHCT**

1. What family values affect CHCT?
2. ...................................................................................................................................................
3. …………………………………………………………………………………………………….…..
4. ……………………………………………………………………………………………………………
5. …………………………………………………………………………………………………………..
6. …………………………………………………………………………………………………………
7. What community values affect CHCT?
8. ...................................................................................................................................................
9. …………………………………………………………………………………………………….…..
10. ……………………………………………………………………………………………………………
11. …………………………………………………………………………………………………………..
12. …………………………………………………………………………………………………………
13. What risks encourage a couple to test for HIV together?
14. ...................................................................................................................................................
15. …………………………………………………………………………………………………….…..
16. ……………………………………………………………………………………………………………
17. …………………………………………………………………………………………………………..
18. …………………………………………………………………………………………………………
19. What are the benefits of CHCT?
20. ...................................................................................................................................................
21. …………………………………………………………………………………………………….…..
22. ……………………………………………………………………………………………………………
23. …………………………………………………………………………………………………………..
24. …………………………………………………………………………………………………………
25. Why would you recommend a couple to go for CHCT?
26. ...................................................................................................................................................
27. …………………………………………………………………………………………………….…..
28. ……………………………………………………………………………………………………………
29. …………………………………………………………………………………………………………..
30. …………………………………………………………………………………………………………

**SECTION D: Structural factors and CHCT**

1. What are the inputs into health care that increase the effectiveness of CHCT?
2. ...................................................................................................................................................
3. …………………………………………………………………………………………………….…..
4. ……………………………………………………………………………………………………………
5. …………………………………………………………………………………………………………..
6. …………………………………………………………………………………………………………

How does the health care system in Uganda influence couples to test for HIV?

(Accessibility, Motivation of health workers, User fees, Availability of HCT supplies, Counseling services, Availability of care sites, Follow ups of those tested, Availability of information about CHCT, Confidentiality)

1. ...................................................................................................................................................
2. …………………………………………………………………………………………………….…..
3. ……………………………………………………………………………………………………………
4. …………………………………………………………………………………………………………..
5. …………………………………………………………………………………………………………

Thank you for participating
