# Supplementary material for: Motivators of couple HIV counseling and testing (CHCT) uptake in a rural setting in Uganda
Source: BMC Public Health. 2017 Jan 23;17:104. doi: 10.1186/s12889-017-4043-z (PMC5259987; doi:10.1186/s12889-017-4043-z)
Supplement: Additional file 2: — Key informant interview and FGD guide (DOC 25 kb) [file 12889_2017_4043_MOESM2_ESM.doc]

**Key informant interview and FGD guide**

1. What has government put in to ensure that couples test for HIV together?
2. Why do couples fail to come for HCT together?
3. What are the benefits of couple HCT that would encourage couples to test for HIV together?
4. What are the risks of HIV infection that would encourage persons to test for HIV?
5. What motivates health workers to influence couples to test for HIV together?
6. How does the family fail a couple to test for HIV together?
7. How does the community fail a couple to take up CHCT?
